# Phase Equilibria of the Fe–Cr–Er Ternary System in the Range 973–1273 K

**DOI:** 10.3390/ma16041705

**Published:** 2023-02-17

**Authors:** Chenbo Li, Yusong Nie, Rong Yin, Jifeng Yang, Lideng Ye, Libin Liu, Ligang Zhang

**Affiliations:** School of Material Science and Engineering, Central South University, Changsha 410083, China

**Keywords:** Fe–Cr–Er system, phase equilibria, solid solubility, isothermal section, oxide dispersion-strengthened steel

## Abstract

Phase relations of the Fe–Cr–Er system in the temperature range 973–1273 K were experimentally investigated using equilibrated alloys. The isothermal sections consisted of 9 single-phase regions, 16 two-phase regions, and 8 three-phase regions at 973 K and 1073 K. At 1273 K, the σ phase disappeared, and liquid appeared. All single phases had a solid solubility range that showed a downward trend with a decrease in temperature. The homogeneity range of the ErFe_12−x_Cr_x_ ternary compound was determined to be x = 1.8–4.5. The more accurate phase relations obtained in this work can better guide the preparation of Fe–Cr–Er alloys in actual production.

## 1. Introduction 

Oxide dispersion-strengthened (ODS) steel, which developed from martensitic and ferritic steels, is the most promising candidate for nuclear reaction cladding [1]. ODS steel is strengthened by dispersing oxide particles, which strongly impede dislocation motion and thereby increase the onset stress of plastic deformation and creep resistance [2,3]. ODS ferritic/martensitic steels containing 9–12 mass% Cr have been developed as fuel cladding material because of their high creep strength at elevated temperatures and adequate resistance to neutron irradiation embrittlement [4,5,6,7,8,9]. The performance of ODS steel largely depends on the particle size and stability of the dispersed oxide nanoparticles [10,11]. Oxide particles containing rare earth elements, especially Y2O3, are the most widely used; however, the concentration of oxide particles containing Y is readily saturated in ODS steel [12]. To further increase the concentration of oxide particles to improve the material properties, it is necessary to add other rare earth elements. Er not only forms oxide particles but can also form magnetic intermetallic compounds with transition metals, which can play a magnetic refrigeration role and prevent the reactor from overheating and causing accidents [13,14,15,16,17,18,19,20]. Therefore, ODS steel containing the Fe–Cr–Er system is a potential nuclear reactor cladding material. 

To study the Fe–Cr–Er system, it is very important to know the thermodynamic information of the ternary system. Phase diagrams are an effective means to intuitively express the relationship between phases in a thermodynamic equilibrium state and are the basic theoretical guidance for the research, development, and design of new materials [21,22,23,24,25,26]. However, the phase diagram information of the Fe–Cr–Er ternary system is still lacking. Only Pan et al. [27] measured its isothermal section at 773 K in 2014. The chemical composition and phase content of multi-component iron-based alloys at different temperatures directly affect macroscopic properties of the material [28,29,30]. Considering that the nuclear reactor is operated at high temperatures, only studying 773 K is far from practical. Therefore, the isothermal section of the Fe–Cr–Er ternary system needs to be completed. In this work, the isothermal sections of the Fe–Cr–Er ternary system at 1273 K, 1073 K, and 973 K were determined by the equilibrium alloy method.

**Figure 1:**
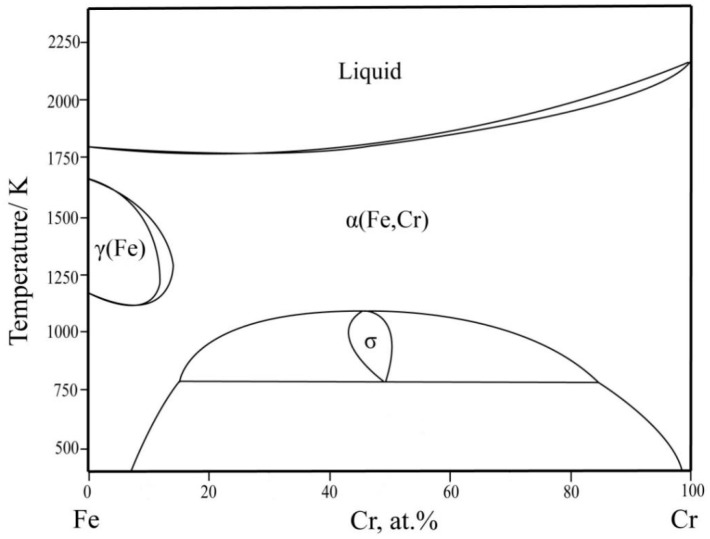
The calculated Fe–Cr phase diagram based on the work of Jacob et al. [31].

The Fe–Cr binary system has been extensively studied as the basis for the study of many engineering materials [31,32,33,34]. The σ phase is the most prominent characteristic phase in the Fe–Cr binary system. Menezes et al. [35] found the existence of the σ phase by X-ray diffraction (XRD) characterization. Bergman et al. [36] analyzed the crystal structure of the σ phase. Andersson et al. [37] performed the first thermodynamic evaluation of the Fe–Cr system. Based on Andersson’s work, Lee [34] modified the model of the liquid phase and improved the Fe–Cr phase diagram. Xiong et al. [38] provided a comprehensive summary of thermodynamic calculations for the Fe–Cr system and updated descriptions concerning the miscibility gap band, Curie temperature, and magnetic moment. Jacob et al. [31] adopted first-principles calculations to re-select the σ phase model and further optimized the Fe–Cr phase diagram, as shown in Figure 1.

**Figure 2:**
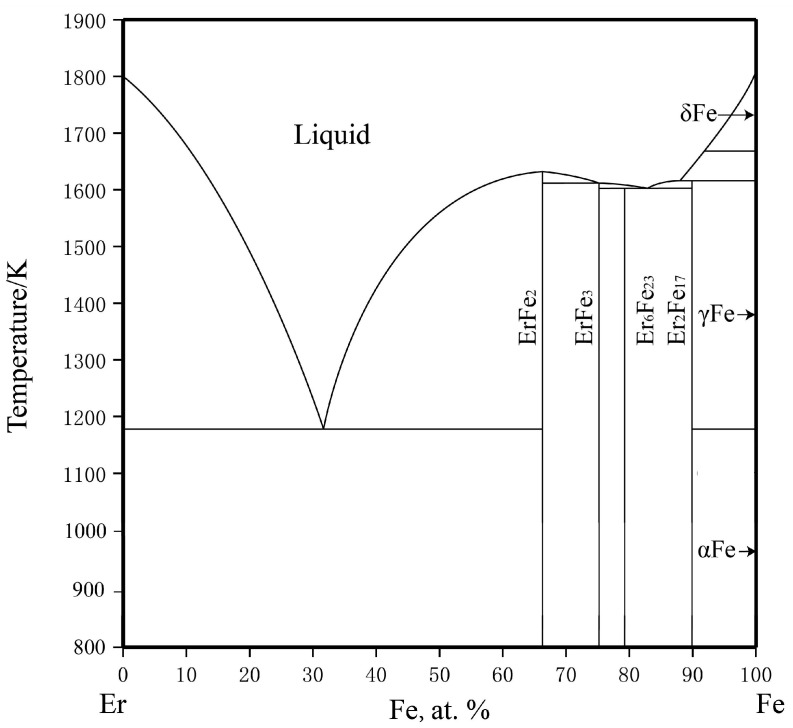
The calculated Er-Fe phase diagram based on the work of Zhou et al. [42].

There are four compounds (ErFe_2_, ErFe_3_, Er_6_Fe_23_, and Er_2_Fe_17_) in the Fe–Er binary system. Meyer [39] obtained the Fe–Er binary phase diagram by thermal analysis, electron probe microanalysis (EPMA), and XRD. Buschow and Goot [40] studied the phase relationship, crystal structure, magnetic properties, and lattice constants of various intermetallic compounds in the Fe–Er system and noticed that Fe and Er show mutual solubility. The Miedema model was employed to calculate the enthalpy of the formation of an intermetallic compound in the Fe–Er system [41]. Recently, Zhou et al. [42] conducted a detailed thermodynamic evaluation of the Fe–Er system, as shown in Figure 2.

**Figure 3:**
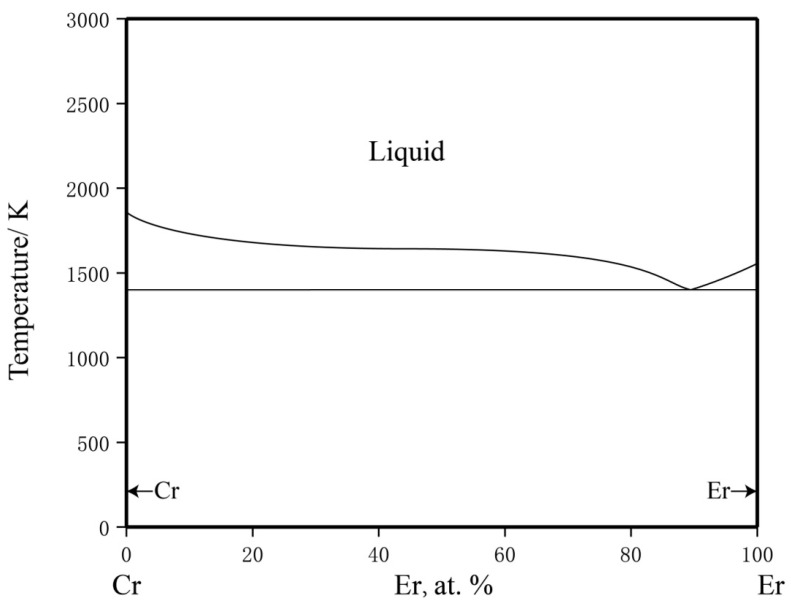
The calculated Cr–Er phase diagram based on the work of Ray et al. [43].

No intermetallic compounds in the Cr–Er binary system have been reported. The calculated phase diagram used in this work adopted the latest thermodynamic parameters reported by Ray et al. [43] in 1996, as shown in Figure 3.

**Figure 4:**
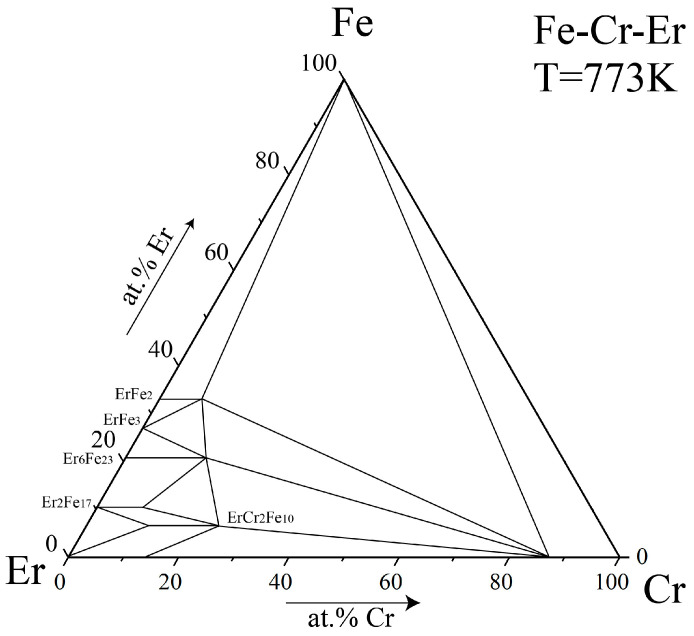
Isothermal section of Fe–Cr–Er ternary system at 773 K by Pan et al. [27].

In the Fe–Cr–Er ternary system, it is generally believed that there are two types of intermetallic compounds: ErCr_12−x_Fe_x_ and Er_3_Cr_29−x_Fe_x_ [44,45]. Stefanski et al. [46] identified ErCr_2_Fe_10_ and studied its structure. On this basis, Bara [47] further discussed the magnetic properties of ErCr_2_Fe_10_. Luo [48] synthesized a series of Er_3_Cr_29−x_Fe_x_ compounds to investigate their structures and magnetic properties by XRD and magnetic measurements. Luo [48] found that all Er_3_Cr_29−x_Fe_x_ compounds crystallized in disordered Th_2_Ni_17_-type structures. By combining the three related binary phase diagrams, Pan et al. [27] measured an isothermal section of the Fe–Cr–Er ternary phase diagram and analyzed the relationship between different phases at 773 K, as shown in Figure 4. Previously reported binary and ternary phases, crystal structures, and lattice parameters are listed in Table 1.

## 2. Materials and Methods

The equilibrium alloy method of static measurement was adopted in this work. Iron rod (99.99 mass%), chromium rod (99.99 mass%), and erbium block (99.99 mass%) were selected as raw materials. Considering the high density of Er, the mass of each alloy sample was designed to be 18 g. The compositions of each alloy are listed in Table 2, Table 3 and Table 4. To prevent the alloy samples from being oxidized, sponge titanium was used as an oxygen absorbent in the arc-melting process. Each sample was melted on a water-cooled copper crucible under a high-purity argon atmosphere. To ensure homogeneity, each sample was remelted at least six times. The prepared alloy samples were sealed in quartz tubes filled with argon as a protective gas and annealed at 973 K and 1073 K for 90 days and at 1273 K for 60 days. After annealing, the alloys were quenched in ice water to retain the high-temperature microstructure.

EPMA (JAXA-8800 R, JEOL, 15 kV, 1 × 10^−8^ A, Tokyo, Japan) equipped with an OXFORD INCA 500 wave-dispersive X-ray spectrometer (WDS, JAXA-8800 R, JEOL, 15 kV, 1 × 10^−8^ A, Tokyo, Japan) was used to detect the microstructure of equilibrated alloys and composition of each phase, including solubility. XRD (Rigaku d-max/2550 VB, Cu K, 40 kV, 250 mA, Tokyo, Japan) was employed to analyze the crystal structure of typical alloys within the scanning range of 10°–90° and speed of 0.133°/s. The data were analyzed by JADE 8.7 software. Backscattered electron (BSE) images of the alloy samples were acquired using a scanning electron microscope (SEM; TESCAN MIRA3 LMH, 15 kV, working distance of 15 mm, Brno, Czech Republic).

## 3. Results

**Figure 5:**
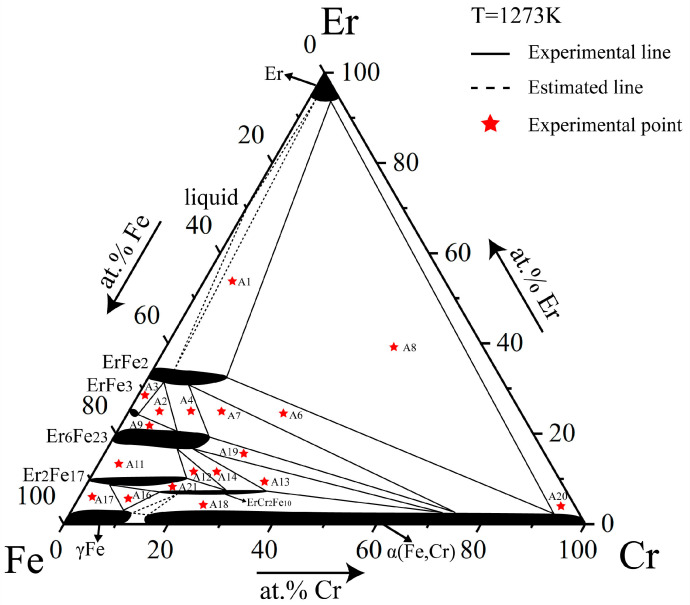
Isothermal section of the Fe–Cr–Er ternary system at 1273 K determined in this work.

The isothermal section of the Fe–Cr–Er system at 1273 K was obtained based on the analysis of typical alloy samples at 1273 K. The maximum solid solution solubilities of Cr were 15.19 at.%, 2.46 at.%, 18.67 at.%, and 18.89 at.% in ErFe_2_, ErFe_3_, Er_6_Fe_23_, and Er_2_Fe_17_, respectively. Only ErCr_2_Fe_10_ was found as a ternary compound at this temperature and could dissolve about 20.43 at.% Cr at most. In addition to the solution of Cr mentioned above, this work found that Er also can dissolve as ErFe_2_, ErFe_3_, Er_6_Fe_23_, and Er_2_Fe_17_. This is a clear difference from the results of Pan et al. [27]. This phenomenon may be due to the influence of the experimental temperature. The Fe–Er binary phase diagram also showed no solid solubility of Er in Fe–Er binary compounds. After repeatedly confirming the accuracy of the experimental data, we speculate that the addition of Cr may affect the solubility of Er in Fe–Er binary compounds. Comparison between this work and published literature [31,39,43] shows that the phase relationships are accurate, except that solubility differs slightly in the relevant binary phase diagrams. This confirmed the reliability of the Fe–Cr–Er isothermal section at 1273 K, shown in Figure 5.

**Figure 6:**
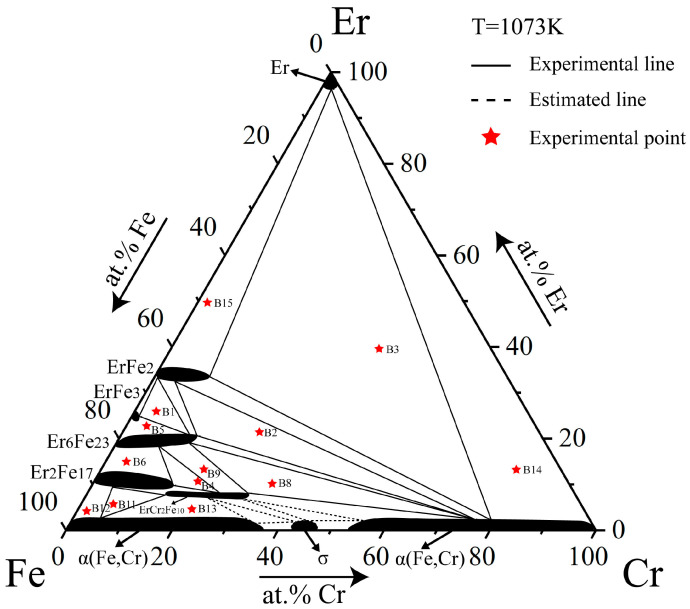
Isothermal section of the Fe–Cr–Er ternary system at 1073 K obtained in this work.

The isothermal section of the Fe–Cr–Er ternary system at 1073 K is similar to that at 1273 K, except that the maximum solid solubilities of ErCr_2_Fe_10_, ErFe_2_, ErFe_3_, Er_6_Fe_23_, Er_2_Fe_17_, and Er are slightly lower. Additionally, α(Fe,Cr) appears at the Fe-enriched corner instead of γ(Fe) at 1073 K. This is consistent with the binary optimized phase diagram, and therefore its appearance is reasonable and in accordance with expectation [31]. The 1073 K isothermal section obtained in this work is shown in Figure 6.

**Figure 7:**
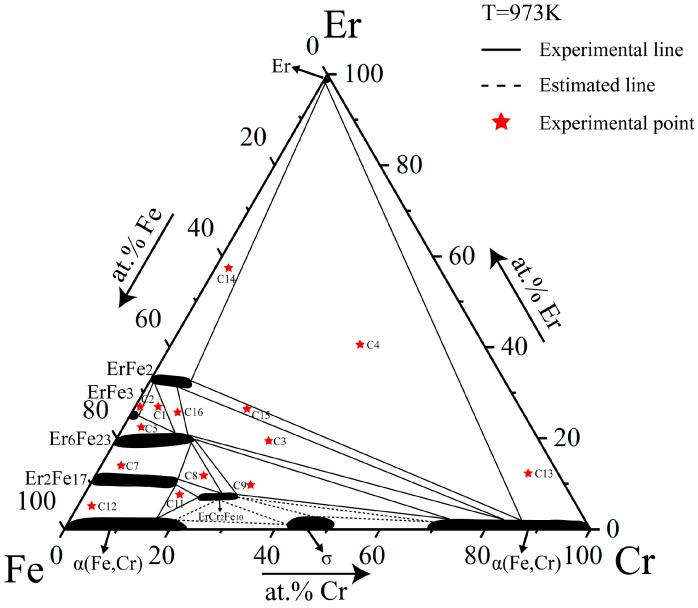
Isothermal section of the Fe–Cr–Er ternary system at 973 K obtained in this work.

The isothermal section of the Fe–Cr–Er ternary system at 973 K was determined based on phase equilibrium data for 13 alloy samples at 973 K, as shown in Figure 7. Eight three-phase regions and 16 two-phase regions were measured. In this isothermal section, there was only one ternary compound, ErCr_2_Fe_10_, and four binary compounds, all having a solid solubility interval. In the region with low Er content, Fe and Cr also formed α(Fe, Cr). The solid solution range of all compounds in this system became narrower than that at 1073 K, so it can be speculated that the solid solution range narrowed as the temperature decreased.

## 4. Discussion

The experimental data obtained from SEM, EPMA, and XRD examination were analyzed to determine the isothermal sections and phase relationships of the Fe–Cr–Er ternary system at 1273 K, 1073 K, and 973 K. In the following context, the phase relations in several key alloys are discussed in detail.

### 4.1. Phase Equilibria at 1273 K

Twenty alloy samples were prepared to determine the isothermal section and phase relationships of the Fe–Cr–Er ternary system at 1273 K. The constituent phases of each alloy sample are listed in Table 2. The nominal composition was set before synthesizing each alloy, and the content of each element in each phase was measured by WDS.

**Figure 8:**
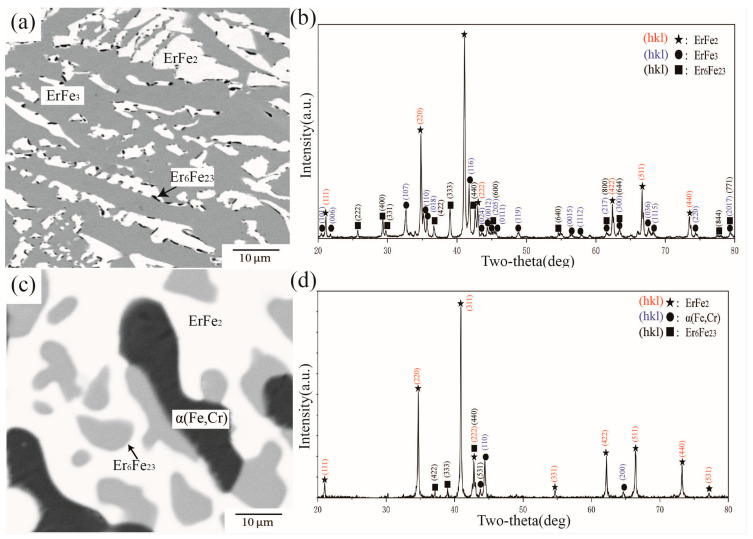
(**a**) BSE image of alloy A2, (**b**) XRD result of alloy A2, (**c**) BSE image of alloy A7, (**d**) XRD result of alloy A7.

Figure 8 presents BSE images and XRD patterns of alloys A2 and A7 annealed at 1273 K. From the observed phase distribution in Figure 8a, there were three different phases in A2. Analysis of the X-ray diffraction pattern in Figure 8b indicated that the phase composition of A2 was the three-phase equilibrium of ErFe_2_ + ErFe_3_ + Er_6_Fe_23_. WDS further showed Cr concentrations of 11.73 at.%, 1.72 at.%, and 3.56 at.% in Er_6_Fe_23_, ErFe_3_, and ErFe_2_, respectively. Compared with alloy A2, shown in Figure 8c,d, it was determined that alloy A7 was located in the three-phase equilibrium region of ErFe2 + α(Fe,Cr) + Er_6_Fe_23_. The WDS results further showed solid solubilities of Cr in Er_6_Fe_23_ and ErFe_2_ of 18.34 at.% and 6.94 at.%, respectively. These two phases can dissolve each other to a large extent between Fe and Cr, which is considered a reasonable phenomenon from the speculation of the Fe–Cr phase diagram.

**Figure 9:**
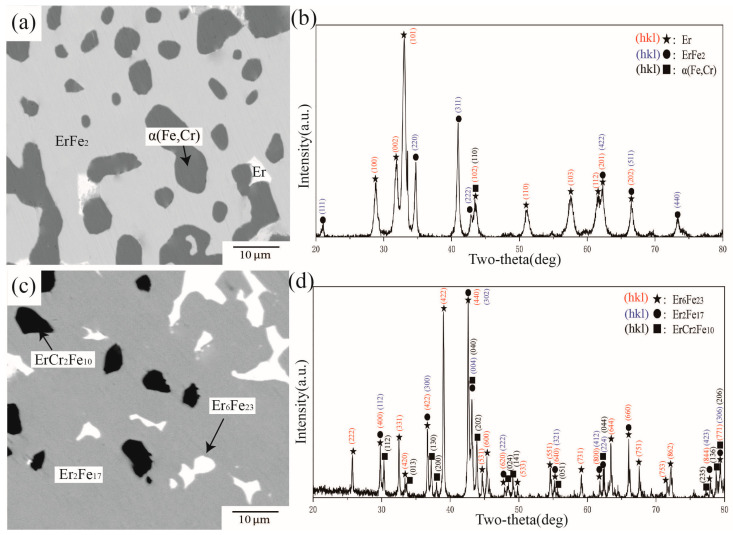
(**a**) BSE image of alloy A8, (**b**) XRD result of alloy A8, (**c**) BSE image of alloy A12, (**d**) XRD result of alloy A12.

BSE images and XRD patterns of alloys A8 and A12 are shown in Figure 9. Figure 9a illustrates the phase composition in alloy A8, which comprises three phases. By comparing standard powder diffraction file (PDF) cards with the diffraction peaks in Figure 9b, it was concluded that A8 was located in a three-phase equilibrium of ErFe_2_ + Er + α(Fe,Cr). The WDS results further showed approximately 14.80 at.% Cr dissolved in ErFe2. Combined with information extracted from XRD, the black area in Figure 9c was identified as the ternary intermetallic compound ErCr_2_Fe_10_. The other two phases with different contrasts are Er_2_Fe_17_ and Er_6_Fe_23_. The WDS results further showed approximately 13.46 at.% Cr dissolved in Er_6_Fe_23_ and 18.32 at.% Cr in Er_2_Fe_17_. As the solitary ternary compound in this system, ErCr_2_Fe_10_ dissolved more Cr and had a certain width in the direction of Er in this work compared with that of Pan et al. [27].

**Figure 10:**
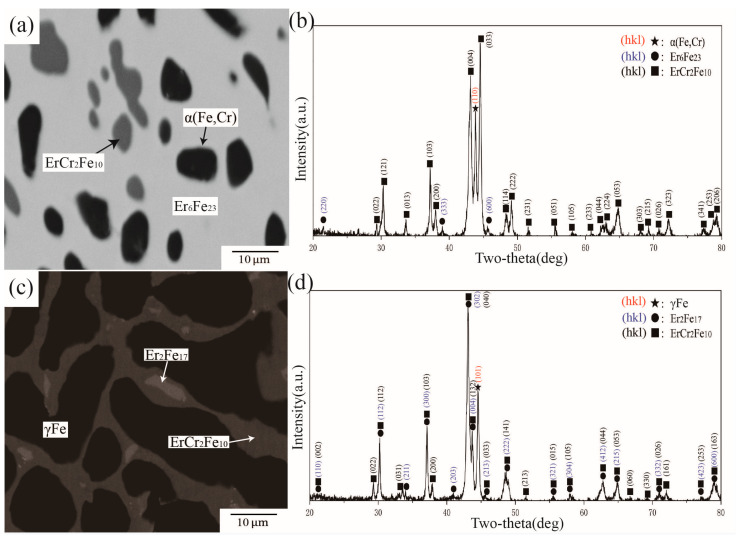
(**a**) BSE image of alloy A13, (**b**) XRD result of alloy A13, (**c**) BSE image of alloy A16, (**d**) XRD result of alloy A16.

BSE images and XRD patterns of alloys A13 and A16 are shown in Figure 10. Figure 10a illustrates the phase composition of alloy A13, which comprises three phases. According to the analysis of characteristic peak positions in Figure 10b, ErCr_2_Fe_10_, Er_6_Fe_23_, and α(Fe,Cr) were determined in A13 and formed a three-phase equilibrium. WDS further determined that approximately 16.75 at.% and 11.90 at.% Cr were dissolved in Er_6_Fe_23_ and ErCr_2_Fe_10_, respectively. Figure 10c,d show BSE images and XRD patterns of A16. Although there is only one characteristic peak of γ(Fe) in the 2θ range from 20° to 80°, the peak exists independently. By combining the law of phase equilibrium and the Fe–Cr phase diagram, A16 was defined in the three-phase area of Er_2_Fe_17_ + ErCr_2_Fe_10_ + γ(Fe). According to WDS data, the maximum atomic percentage of Cr in γ(Fe) was 10.02 at 1273 K in A16. The solubility of Cr in γ(Fe) conforms to the description of Jacob et al. [31].

### 4.2. Phase Equilibria at 1073 K

Fourteen alloy samples were prepared to determine the isothermal section and phase relationships of the Fe–Cr–Er ternary system at 1073 K. The constituent phases of each alloy sample are listed in Table 3.

**Figure 11:**
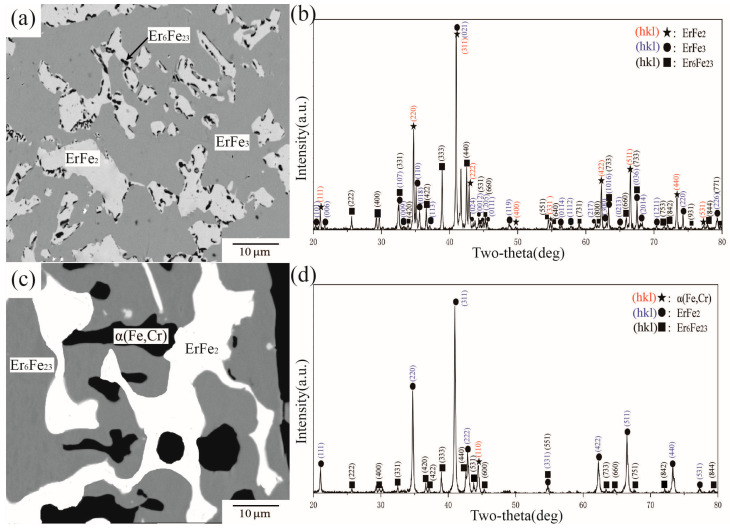
(**a**) BSE image of alloy B1, (**b**) XRD result of alloy B1, (**c**) BSE image of alloy B2, (**d**) XRD result of alloy B2.

Both B1 and B2 had three phases based on the BSE images (Figure 11a,c). According to the XRD and WDS results, all phases are already identified. B1 and B2 are located in three-phase equilibria of ErFe_2_ + Er_6_Fe_23_ + ErFe_3_ and ErFe_2_ + Er_6_Fe_23_ + α(Fe,Cr), respectively. Cr was present at 13.49 at.% and 14.85 at.% in the compound Er_6_Fe_23_ in B1 and B2. There was less than 18.67 at.% Cr, which is the maximum solid solution solubility of Cr at 1273 K. As the temperature decreased by 200 K, the solid solubility of Cr in Er_6_Fe_23_ showed a significant declining trend.

**Figure 12:**
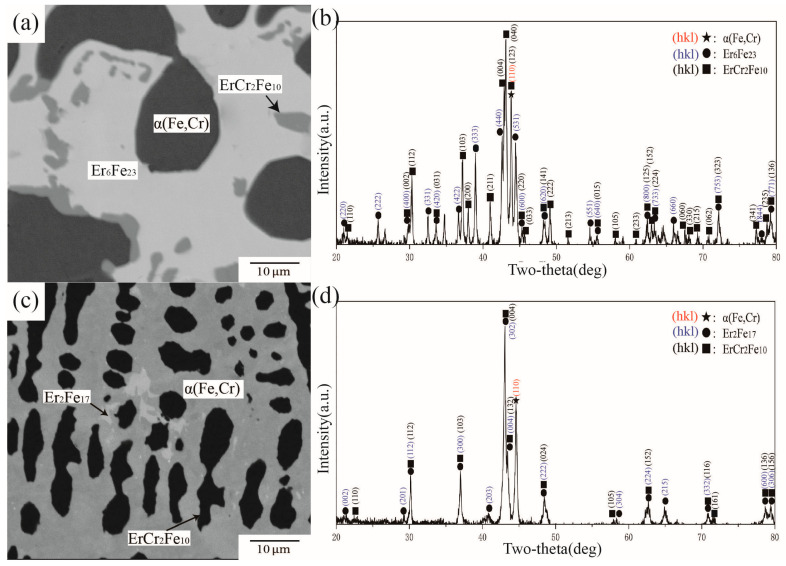
(**a**) BSE image of alloy B8, (**b**) XRD result of alloy B8, (**c**) BSE image of alloy B11, (**d**) XRD result of alloy B11.

BSE images of alloy samples B8 and B11 are shown in Figure 12a,c. The gray phase in B8 and dark gray phase in B11 have the same atomic ratios of ErCr2Fe10, and the XRD characteristic peaks of ErCr_2_Fe_10_ (shown in Figure 12b,d) are basically consistent with the PDF card of the known compound, ErCr_2_Fe_10_. To sum up, ErCr_2_Fe_10_ does not disappear with the decrease in temperature and is a phase that can exist stably at low temperatures. Noticeably, γ(Fe) was replaced by α(Fe,Cr) in the Fe-enriched corner. This is reasonable and consistent with the Fe–Cr phase diagram.

**Figure 13:**
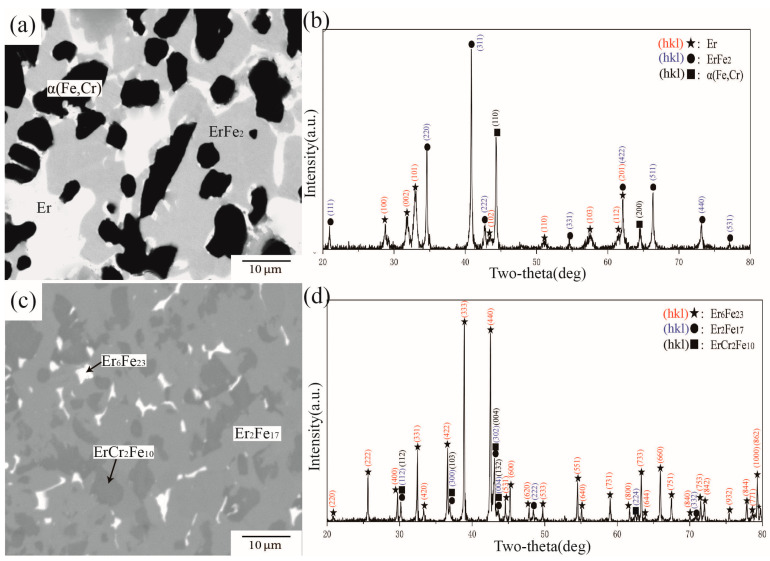
(**a**) BSE image of alloy B3, (**b**) XRD result of alloy B3, (**c**) BSE image of alloy B7, (**d**) XRD result of alloy B7.

Figure 13 shows BSE images and XRD patterns of alloys B3 and B7. By combining the data shown in Figure 13a,b, the ErFe_2_, Er, and α(Fe,Cr) phases were found in B3, indicating a three-phase equilibrium of ErFe_2_ + Er + α(Fe,Cr). The WDS results further validate these phases and indicate a solid solubility of Cr of 10.48 at.% in ErFe_2_. In Figure 13a, the uniform distribution of Er in the area also proved that it precipitated in a later stage. This can be used to judge whether the sample was in equilibrium. Compared with alloy B3, shown in Figure 13c,d, it can be determined that alloy B7 was located in the three-phase equilibrium region of Er_2_Fe_17_ + Er_6_Fe_23_ + ErCr_2_Fe_10_. The WDS results further showed that approximately 8.35 at.% Cr was dissolved in Er_6_Fe_23_ and approximately 17.77 at.% of Cr was dissolved in Er_2_Fe_17_.

### 4.3. Phase Equilibria at 973 K

Thirteen alloy samples were prepared to determine the isothermal section and phase relationships of the Fe–Cr–Er ternary system at 973 K. The constituent phases of each alloy sample are listed in Table 4.

**Figure 14:**
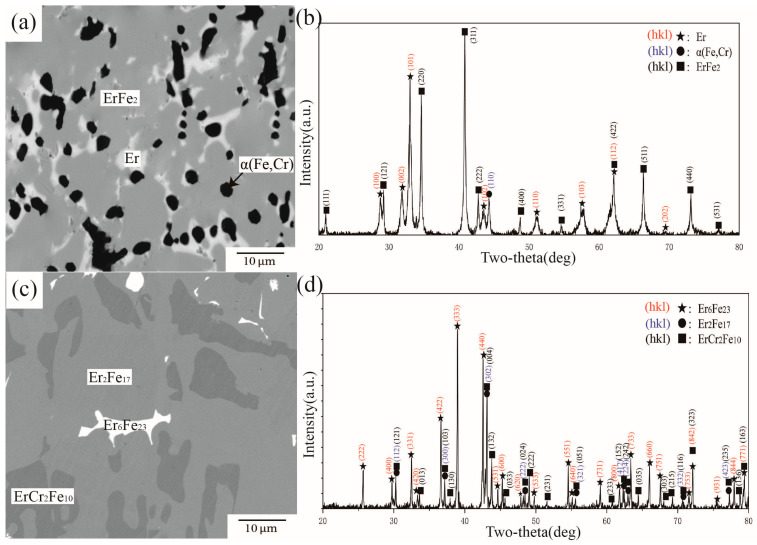
(**a**) BSE image of alloy C4, (**b**) XRD result of alloy C4, (**c**) BSE image of alloy C8, (**d**) XRD result of alloy C8.

There are three obvious contrasts in the BSE images of alloy samples C4 and C8, as shown in Figure 14a,c. By comparing standard PDF cards and the characteristic peaks in Figure 14b,d, C4 and C8 belong to the three-phase equilibria ErFe_2_ + Er + α(Fe,Cr) and Er_2_Fe_17_ + Er_6_Fe_23_ + ErCr_2_Fe_10_, respectively. By analyzing WDS data, the maximum solid solubilities of Cr in ErFe_2_ and Er_2_Fe_17_ were 7.50 at.% and 16.23 at.%, respectively. The solubility of Cr in Fe–Er binary compounds was significantly reduced compared with that at 1073 K and 1273 K.

**Figure 15:**
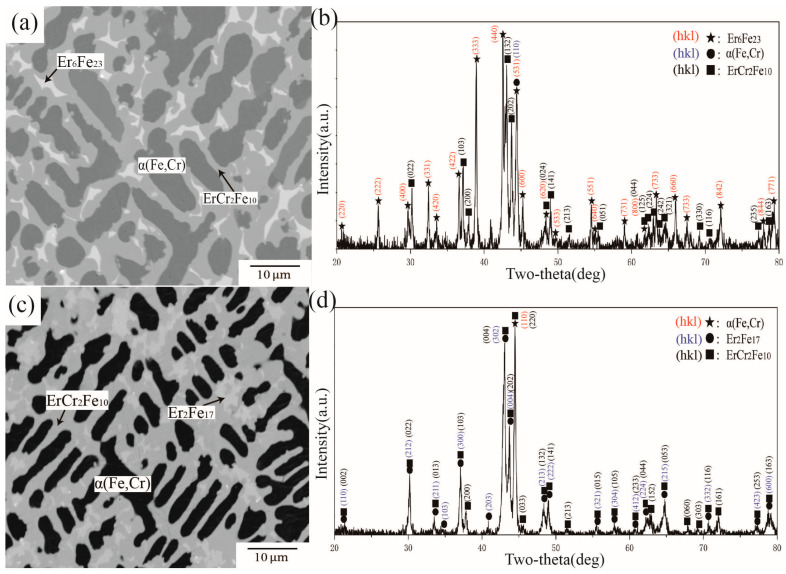
(**a**) BSE image of alloy C9, (**b**) XRD result of alloy C9, (**c**) BSE image of alloy C11, (**d**) XRD result of alloy C11.

There are three obvious contrasts in the BSE images of alloy samples C9 and C11, as shown in Figure 15a,c. By comparing standard PDF cards and the characteristic peaks in Figure 15b,d, C9 and C11 belong to three-phase equilibrium ErFe_2_ + Er + α(Fe,Cr) and Er_2_Fe_17_ + Er_6_Fe_23_ + ErCr_2_Fe_10_, respectively. By analyzing WDS data obtained, the maximum solid solubilities of Cr in ErFe_2_ and Er_2_Fe_17_ were 7.50 at.% and 16.23 at.%, respectively. The solubility of Cr in Fe–Er binary compounds is significantly reduced compared with that at 1073 K and 1273 K.

## 5. Conclusions

In this work, the phase relationships of the Fe–Cr–Er ternary system at 1273 K, 1073 K, and 973 K results were systematically studied by combining data from WDS, XRD, and SEM measurements. There are nine single-phase regions, 16 two-phase regions, and eight three-phase regions at 973 K and 1073 K. At 1273 K, the σ phase disappeared, and liquid appeared. Phase equilibrium relationships are similar in the different isothermal sections studied in this work. Although the solid solubility of Fe–Er binary compounds is not completely consistent with the marginal binary phase diagram due to the influence of Cr, the phase relationship is basically similar. The maximum solid solubilities of Cr in ErFe_2_, ErFe_3_, Er_6_Fe_23_, and Er_2_Fe_17_ were 15.19 at.%, 2.47 at.%, 18.67 at.%, and 18.89 at.% at 1273 K, respectively. These values reduced to 10.70 at.%, 1.81 at.%, 14.85 at.%, and 17.77 at.% at 1073 K and continued to decrease to 8.12 at.%, 1.79 at.%, 14.33 at.%, and 16.23 at.% at 973 K, respectively. ErCr_2_Fe_10_ existed in all three isothermal sections, from which it can be determined that it forms a stable ternary compound from 973 K to 1273 K. At 1273 K, Cr had the strongest solubility in ErCr_2_Fe_10_, and the solid solubility ranged from Fe10.2Cr1.8Er to Fe7.5Cr4.5Er. Accurate determination of solubility range can help to analyze the existence and movement behavior of elements in phases. These isothermal sections at 1273 K, 1073 K, and 973 K provide the possibility of obtaining a thermodynamic description using the CALPHAD (CALculation of PHAse Diagrams) method. Thermodynamic optimization can calculate the phase relationship of the system at any temperature, combine the physical, chemical, and mechanical properties of the phase, and guide alloy composition design in actual production to obtain a desired material.

## Figures and Tables

**Table 1:** Experimental and literature data on crystal structures and lattice parameters of the solid phases in the Fe–Cr–Er system.

Phase	Phase Prototype	Space Group		Lattice Parameters (nm)		Reference
			a	b	c	
αFe	cI2	Im3¯m	0.2862	0.2862	0.2862	[1]
γFe	cF4	$Fm\bar{3}m$	0.3613	0.3613	0.3613	[1]
Cr	cI2	Im3¯m	0.2910	0.2910	0.2910	[2]
Er	hP2	P6_3_/mmc	0.3555	0.3555	0.5584	[15]
σ	tP30	P4_2_/mmm	0.8802	0.8802	0.4548	[35]
ErFe_2_	cF24	$\mathrm{Fd}\bar{3}m$	0.7316	0.7316	0.7316	[39]
ErFe_3_	hR36	$R\bar{3}m$	0.5086	0.5086	24464	[39]
Er_6_Fe_23_	cF116	$Fm\bar{3}m$	1.1994	1.1994	1.1994	[39]
Er_2_Fe_17_	hP38	P6_3_/mmc	0.8444	0.8444	0.8268	[39]
ErCr_2_Fe_10_	t/26	I4/mmm	0.8534	0.8534	0.4761	[46]
Er_3_Cr_12-x_Fe_x_	hP80	P63/mmc	0.8416	0.8416	0.8325	[48]

**Table 2:** Constituent phases and compositions in the annealed Fe–Cr–Er alloys at 1273 K for 60 days.

Alloys No.	Nominal Composition (at.%)			Experimental Results (at.%)			Phase Determination
	Fe	Cr	Er	Fe	Cr	Er	
A1	40	5	55	46.92	15.26	37.82	ErFe_2_
				1.57	0.55	97.88	Er
A2	70	5	25	67.56	11.73	20.71	Er_6_Fe_23_
				73.8	1.72	24.48	ErFe_3_
				63.44	3.56	33	ErFe_2_
A3	70	2	28	63.04	2.35	34.61	ErFe_2_
				74.01	1.07	24.92	ErFe_3_
A4	65	10	25	57.89	11.72	30.39	ErFe_2_
				61.67	16.87	21.46	Er_6_Fe_23_
A5	59	11	30	60.15	10.08	29.77	ErFe_2_
A6	53	25	22	11.15	86.07	2.78	α(Fe,Cr)
				62.15	7.82	30.03	ErFe_2_
A7	60	20	20	61.72	18.34	19.94	Er_6_Fe_23_
				61.89	6.94	31.17	ErFe_2_
				21.56	75.93	2.51	α(Fe,Cr)
A8	20	40	40	52.53	14.8	32.67	ErFe_2_
				1.21	4.48	94.31	Er
				5.03	92.03	2.94	α(Fe,Cr)
A9	70	8	22	73.92	2.47	24.61	ErFe_3_
				70.18	8.69	21.13	Er_6_Fe_23_
A10	73	10	18	72.43	9.7	17.87	Er_6_Fe_23_
A11	80	5	15	68.86	10.11	21.03	Er_6_Fe_23_
				85.61	3.32	11.07	Er_2_Fe_17_
A12	68	20	12	64.22	28	7.78	ErCr_2_Fe_10_
				69.81	13.46	16.73	Er_6_Fe_23_
				71.95	18.32	9.73	Er_2_Fe_17_
A13	56	35	9	66.75	16.75	16.5	Er_6_Fe_23_
				57.43	34.99	7.58	ErCr_2_Fe_10_
				26.63	71.08	2.29	α(Fe,Cr)
A14	65	25	10	67.74	15.76	16.5	Er_6_Fe_23_
				61.81	30.86	7.33	ErCr_2_Fe_10_
A15	83	5	12	84.16	4.61	11.23	Er_2_Fe_17_
A16	85	10	5	86.72	4.57	8.71	Er_2_Fe_17_
				78.11	15.02	6.87	ErCr_2_Fe_10_
				87	10.02	2.98	γFe
A17	93	2	5	94.74	2.28	2.98	γFe
				88.78	2.28	8.94	Er_2_Fe_17_
A18	71	25	4	68.35	24.26	7.39	ErCr_2_Fe_10_
				80.02	17.88	2.1	α(Fe,Cr)
A19	57	27	16	63.88	18.24	17.87	Er_6_Fe_23_
				23.67	74.08	2.25	α(Fe,Cr)
A20	2	92	6	2.85	95.31	1.83	α(Fe,Cr)
				0.65	5.41	93.94	Er

**Table 3:** Constituent phases and compositions in the annealed Fe–Cr–Er alloys at 1073 K for 90 days.

Alloys No.	Nominal Composition (at.%)			Experimental Results (at.%)			Phase Determination
	Fe	Cr	Er	Fe	Cr	Er	
B1	70	5	25	64.6	13.49	20.01	Er_6_Fe_23_
				73.26	1.81	24.93	ErFe_3_
				65.65	1.04	33.31	ErFe_2_
B2	50	30	20	64.6	14.85	20.55	Er_6_Fe_23_
				62.87	4.54	32.59	ErFe_2_
				21.71	75.74	2.55	α(Fe,Cr)
B3	20	40	40	55.96	10.48	33.56	ErFe_2_
				1.42	2.45	96	Er
				17.66	80.11	2.23	α(Fe,Cr)
B4	70	20	10	66.82	25.09	8.09	ErCr_2_Fe_10_
				74.93	15.22	9.85	Er_2_Fe_17_
				72.94	8.56	18.5	Er_6_Fe_23_
B5	73	4	23	72.43	6.34	21.22	Er_6_Fe_23_
				74.37	1.44	24.19	ErFe_3_
B6	80	5	15	77.55	4.3	18.15	Er_6_Fe_23_
				82.91	4.2	12.89	Er_2_Fe_17_
B7	68	20	12	66.4	25.71	7.89	ErCr_2_Fe_10_
				73.26	8.35	18.39	Er_6_Fe_23_
				72.43	17.77	9.8	Er_2_Fe_17_
B8	56	35	9	66.3	14.17	19.53	Er_6_Fe_23_
				61.13	30.9	7.97	ErCr_2_Fe_10_
				22.69	74.92	2.39	α(Fe,Cr)
B9	67	20	13	68.81	12.45	18.75	Er_6_Fe_23_
				64.93	27.27	7.8	ErCr_2_Fe_10_
B10	83	5	12	84.25	4.68	11.07	Er_2_Fe_17_
B11	90	5	5	85.98	4.8	9.22	Er_2_Fe_17_
				77.01	15.38	7.61	ErCr_2_Fe_10_
				91.43	5.71	2.85	α(Fe,Cr)
B12	93	2	5	95.07	2.08	2.85	α(Fe,Cr)
				88.02	1.78	10.2	Er_2_Fe_17_
B13	75	20	5	72.04	20.61	7.35	ErCr_2_Fe_10_
				76.29	20.71	3	α(Fe,Cr)
B14	8	80	12	7.99	90.06	1.95	α(Fe,Cr)
				0.39	2.73	96.87	Er
B15	48	2	50	2.16	1.12	96.72	Er
				61.05	3.15	35.8	ErFe_2_

**Table 4:** Constituent phases and compositions in the annealed Fe–Cr–Er alloys at 973 K for 90 days.

Alloys No.	Nominal Composition (at.%)			Experimental Results (at.%)			Phase Determination
	Fe	Cr	Er	Fe	Cr	Er	
C1	70	5	25	68.13	11.09	20.78	Er_6_Fe_23_
				72.92	2.21	24.87	ErFe_3_
				67.08	1.02	31.9	ErFe_2_
C2	72	1	27	67.31	0.51	32.18	ErFe_2_
				73.04	1.11	25.86	ErFe_3_
C3	50	30	20	66.15	13.21	20.64	Er_6_Fe_23_
				63.24	5.7	31.05	ErFe_2_
				15.39	82.75	1.85	α(Fe,Cr)
C4	25	35	40	59.15	7.5	33.35	ErFe_2_
				1.41	0.77	97.82	Er
				12.21	85.66	2.13	α(Fe,Cr)
C5	75	3	22	74.32	1.65	24.03	ErFe_3_
				75.57	3.63	20.8	Er_6_Fe_23_
C6	73	10	18	71.59	10.87	17.54	Er_6_Fe_23_
C7	80	5	15	78.65	3.78	17.56	Er_6_Fe_23_
				84.13	3.64	12.22	Er_2_Fe_17_
C8	68	20	12	72.84	16.22	10.94	ErCr_2_Fe_10_
				67.46	13.73	18.81	Er_6_Fe_23_
				73.48	16.23	10.28	Er_2_Fe_17_
C9	60	30	10	67.06	13.28	19.66	Er_6_Fe_23_
				63.62	28.64	7.74	ErCr_2_Fe_10_
				25.67	72.32	2.01	α(Fe,Cr)
C10	83	5	12	83.43	5.19	11.38	Er_2_Fe_17_
C11	74	18	8	73.91	16.56	9.53	Er_2_Fe_17_
				71.16	21.84	7	ErCr_2_Fe_10_
				80.77	16.58	2.65	α(Fe,Cr)
C12	93	2	5	94.9	2.71	2.39	α(Fe,Cr)
				88.51	1.93	9.55	Er_2_Fe_17_
C13	5	83	12	6.65	91.79	1.56	α(Fe,Cr)
				0.47	1.29	98.24	Er

## Data Availability

Not applicable.
